# 772 Impact of Clinical Course on Psychosocial Outcomes in Adult Burn Survivors

**DOI:** 10.1093/jbcr/irac012.325

**Published:** 2022-03-23

**Authors:** Erin E Ross, Rachel A Colbath, Jeremy Yu, Naikhoba Munabi, Justin Gillenwater, Haig A Yenikomshian

**Affiliations:** Keck School of Medicine, University of Southern California, Los Angeles, California; Keck School of Medicine of USC, Carlsbad, California; University of Southern California, Los Angeles, California; Keck school of medicine of USC, Los Angeles, California; USC/LAC+USC Medical Center, Los Angeles, California; USC/LAC+USC Medical Center, Los Angeles, California

## Abstract

**Introduction:**

There has been conflicting data on the relationship between burn severity and psychological outcome. The present study aims to characterize the baseline psychosocial disposition of adults attending outpatient burn clinic at a large county hospital, as well as the impact of clinical course on self-reported psychosocial well-being.

**Methods:**

Adult patients attending outpatient burn clinic completed survey questions from National Institutes of Health (NIH) Patient-Reported Outcomes Measurement Information System (PROMIS) Managing Emotions (ME) and Managing Social Interactions (MSI). Sociodemographic variables collected from surveys and retrospective chart review included age, ethnicity, zip code, employment, and marital status. Clinical variables included total body surface area burned (TBSA), initial hospital length of stay (LOS), surgical history, and number of days since injury. US census data based on patient’s home zip code was used to estimate percent of community living below US poverty level. Scores on ME and MSI were compared to the population mean by one-sample T-test. TBSA, hospital LOS, surgery, and days since burn were separately evaluated for associations with ME and MSI scores by Tobit regression while adjusting for sociodemographic variables.

**Results:**

71 patients completed surveys. Compared to the general US population mean score of 50.0, had lower scores in MSI (mean=48.0, p=.041) but not significantly different ME scores (mean=50.9, p=.394). While controlling for age, ethnicity, marital status, employment status, and neighborhood poverty, increased LOS was associated with lower scores on both ME and MSI scores (Table 1); the associations of TBSA, surgical

**Conclusions:**

Burn patients may experience difficulty in getting support after burn injury. This challenge may be increased in patients with more extensive debilitation after injury, as increased duration of hospitalization worsened self-efficacy for managing emotions and social interactions. Additionally, prolonged hospitalization may remove patients from their normal support systems.

